# Effect of colorectal cancer screening on colorectal cancer surgery outcomes: nationwide cohort study

**DOI:** 10.1093/bjsopen/zrae027

**Published:** 2024-03-19

**Authors:** Jannie Dressler, Sisse H Njor, Morten Rasmussen, Lars N Jørgensen

**Affiliations:** Digestive Disease Centre, Bispebjerg Hospital, University of Copenhagen, Copenhagen, Denmark; Research Clinic for Cancer Screening, Randers Regional Hospital, Randers, Denmark; Department of Clinical Medicine, Aarhus University, Aarhus, Denmark; Digestive Disease Centre, Bispebjerg Hospital, University of Copenhagen, Copenhagen, Denmark; Digestive Disease Centre, Bispebjerg Hospital, University of Copenhagen, Copenhagen, Denmark; Department of Clinical Medicine, University of Copenhagen, Copenhagen, Denmark

## Abstract

**Background:**

National colorectal cancer screening commenced in Denmark in 2014. Little is known about the effects of organized colorectal cancer screening on intraoperative and postoperative events. The aim of this nationwide cohort study was to evaluate the difference in intraoperative and postoperative outcomes between patients with screen-detected colorectal cancer and non-screen-detected colorectal cancer within the first 90 days after surgery.

**Methods:**

National register data were collected for Danish residents diagnosed with colorectal cancer between January 2014 and March 2018. Outcomes for the two cohorts were reported as relative risk or weighted mean difference. Intraoperative outcomes were blood loss, blood transfusion, tumour perforation, and organ lesion. Postoperative outcomes were complications (surgical and non-surgical) and 90-day mortality. Discrete data estimates were calculated from a general linear model. Analyses were adjusted for potential healthy user bias with respect to sex, age, location of the cancer (colon/rectum), and Charlson co-morbidity index.

**Results:**

In total, 10 606 patients were included. Compared with patients in the non-screen-detected colorectal cancer group (4497 patients), patients in the screen-detected colorectal cancer group (6109 patients) had reduced intraoperative blood loss (−52 mL, 95% c.i. −67 to −37, *P* < 0.001), a shorter duration of hospitalization (−2.3 days, 95% c.i. −2.8 to −1.8, *P* < 0.001), and reduced rates of intraoperative organ lesion (0.76, 95% c.i. 0.59 to 0.99, *P* = 0.042), surgical complications (0.79, 95% c.i. 0.73 to 0.87, *P* < 0.001), non-surgical complications (0.68, 95% c.i. 0.60 to 0.78, *P* < 0.001), and 90-day mortality (0.29, 95% c.i. 0.21 to 0.39, *P* < 0.001).

**Conclusion:**

In comparison with non-screen-detected colorectal cancer, surgery for screen-detected colorectal cancer remains associated with improvement in several intraoperative and early postoperative outcomes after considering healthy user bias.

## Teaser

A national register-based cohort study finding that patients with screen-detected colorectal cancer have fewer intraoperative and postoperative complications, a lower 90-day mortality rate, and shorter hospitalization, in Denmark, during the first round of the national colorectal cancer screening programme.

## Introduction

Colorectal cancer (CRC) represents a substantial health burden, reflected by a global mortality of almost 1 million in 2020^1^. In 2010, the European Union (EU) recommended its member states to implement nationwide, organized, stool sample-based screening for CRC^2^. A national CRC screening programme was implemented in Denmark in 2014 after the EU recommendations. Screening is offered to all Danish residents aged between 50 and 74 years. Every second year, a letter of invitation containing a stool sample kit is sent to each of these residents and, after collection, the stool sample is sent to a laboratory for an occult blood faecal immunochemical test (FIT); if a test is positive, the resident is invited to undergo a colonoscopy^3^. The objective of the screening programme is early detection of CRC and therefore improvement in prognosis after treatment. Moreover, the objective is the prevention of CRC by detection, monitoring, and removal of precancerous lesions^4–6^. The participation rate in Denmark has been around 60% since the implementation of the screening programme. The rate of participants undergoing colonoscopy after a positive FIT is approximately 90%^7^.

It is well established that CRC screening leads to a lower UICC stage at the time of diagnosis^8–14^. There is controversy as to the clinical effect of screening, because not all studies report less invasive surgical interventions in patients with screen-detected colorectal cancer (SD-CRC) compared with non-screen-detected colorectal cancer (NSD-CRC)^15–18^. There is evidence that the level of surgical invasiveness correlates with the rate of surgical and non-surgical complications^19–21^. Only a few studies have investigated the effect of systematic screening on complications after resection of CRC and the results were inconsistent^15–18^. Moreover, two of the studies^15,18^ only included a few patients and two did not report any attempts to overcome healthy user bias^16,17^. Healthy user bias represents the phenomenon that healthy people are more likely to exhibit positive health behaviour, such as participating in screening. The evidence in this area is sparse and not conclusive.

The aim of this study was to evaluate the difference between patients with SD-CRC and NSD-CRC in intraoperative and postoperative outcomes associated with surgical treatment for CRC. It was hypothesized that SD-CRC was associated with an optimized surgical course in comparison with NSD-CRC.

## Methods

This was a nationwide, register-based, retrospective cohort study of patients diagnosed with CRC that aimed to investigate differences in surgical outcomes between patients with SD-CRC and NSD-CRC.

This manuscript was written in accordance with the STROBE guidelines^22^.

### Population

Data were extracted from the Danish Colorectal Cancer Screening (DCCS) database and the Danish Colorectal Cancer Group (DCCG) database^23–25^. The DCCG database included patient, disease, and tumour characteristics in combination with intraoperative and postoperative parameters. These included date of diagnosis, age, sex, Charlson co-morbidity index (CCI), smoking status, alcohol consumption, BMI, UICC stage, tumour location, tumour histology, and surgical priority (elective or emergency). Data entry into the DCCG database is mandatory for all Danish surgeons and pathologists as part of the clinical routine during diagnostics and treatment of CRC. Data in the DCCS database originate from the screening programme administrative system, the National Patient Registry, and the National Pathology Database^25,26^. Data from the DCCS database provided the date of invitation, the date of FIT analysis, the FIT analysis result, and the date and results of colonoscopy. The DCCG and DCCS databases have undergone validity studies that have reported validity of 95% and 97% respectively^27,28^.

The population was defined as Danish residents aged 50–75 years and diagnosed with CRC between 1 January 2014 and 31 March 2018. This interval represented the first round of CRC screening in Denmark. A small proportion of residents in the first screening round had already turned 75 years when they received their invitation, because invitations were sent out around their 75th birthday and not by the month of birth, which was the case for everyone else. These residents were included in the study. The SD-CRC group comprised patients who completed screening and underwent a colonoscopy and were diagnosed with CRC within 6 months after a positive FIT (greater than or equal to 100 ng haemoglobin/mL buffer, corresponding to greater than or equal to 20 µg haemoglobin/g faeces)^8^. The NSD-CRC group comprised patients with CRC who did not submit a stool sample for FIT, who did not undergo a follow-up colonoscopy after a positive FIT, or who received a screening invitation after being diagnosed with CRC.

To avoid length bias, patients with interval- or post-colonoscopy CRC were excluded. Interval cancer was defined as CRC diagnosed after a negative FIT and before the next round of screening. Post-colonoscopy cancer was defined as patients diagnosed after a negative screening colonoscopy. Length bias is a selection bias and found in studies of cancer screening. It represents the phenomenon that screening is more likely to detect slowly progressing tumours. Tumours detected between screening rounds are thus more likely to present with aggressive growth^29^. Patients with synchronous and metachronous CRC were also excluded.

### Outcomes

Intraoperative outcomes were blood loss, blood transfusion, tumour perforation, and organ lesion. The postoperative observation interval was 90 days or until death within 90 days. Postoperative surgical complications were directly related to the surgery. The rates of bleeding, anastomotic leakage (including grading)^30^, bowel obstruction, fascial dehiscence, superficial wound infection, and intra-abdominal abscess were analysed separately. Postoperative non-surgical complications were indirectly related to the surgery and occurred in remote organs (for example stroke, pneumonia, or heart failure). The definition of complications relied on standardized definitions by the DCCG. Surgical complications were postoperative events in anatomic proximity of the wound. Non-surgical complications were medical events without direct proximity to the surgical wound. Mortality was reported separately.

### Statistical analyses

For comparison of the outcomes between patients with SD-CRC and NSD-CRC, a multivariable log-binominal generalized linear regression model was used to estimate relative risks (RRs) with 95% confidence intervals for all dichotomous data. Discrete data were analysed with a general linear model regression. The analyses were adjusted for sex, 5-year age group, location of the cancer (colon/rectum), and CCI to consider healthy user bias. Tumours in the rectum were defined as tumours located within 15 cm of the anal verge. It was not possible to adjust for the UICC stage in some of the analyses, as the residuals in those analyses would not have been linear, thus violating the conditions for a regression model.

Sub-analyses were conducted for 90-day mortality, duration of hospitalization, and rates of surgical- and non-surgical complications after stratification for UICC stage. Moreover, cancer location-stratified analyses were performed for intraoperative outcomes and surgical- and non-surgical complications. *P* < 0.050 was considered statistically significant. All analyses were performed in SAS Enterprise Guide 7.1^31^.

### Ethics

According to Danish law, approval was not required from the local Ethics Committee for registry-based research. Approval of the study was obtained from the Danish Data Protection Agency (VD-2018-128).

## Results

### Background characteristics

**Fig. 1:**
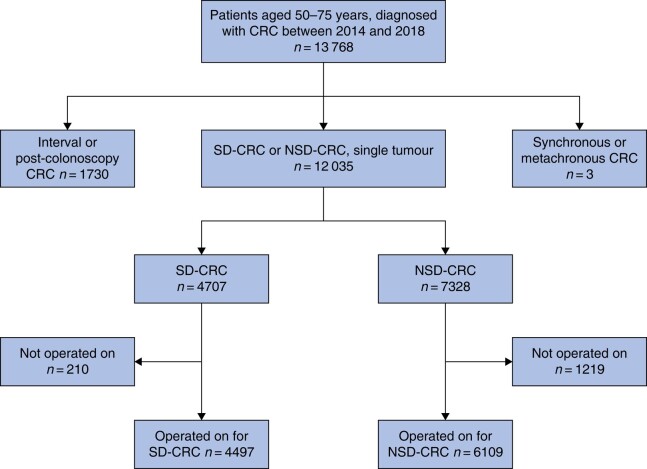
Consort diagram. Patient inclusion

CRC, colorectal cancer; SD-CRC, screen-detected colorectal cancer; NSD-CRC, non-screen-detected colorectal cancer.

A total of 10 606 patients—4497 with SD-CRC and 6109 with NSD-CRC—were included in the study (*Fig. 1*). The distribution of patients with colon and rectal cancer was 7100 and 3506 respectively. The distribution of the two patient groups with regard to patient and disease characteristics was uneven (*Table 1*). Patients with SD-CRC were characterized by higher age, lower CCI, less smoking, more moderate alcohol consumption, higher BMI, lower UICC stage, a higher rate of colon cancer, and a lower rate of emergency surgery.

**Table 1:** Patient and tumour characteristics

	Screen-detected colorectal cancer (*n* = 4497)	Non-screen-detected colorectal cancer (*n* = 6109)	P*
**Age (years)**			<0.001
50–<55	333 (7.4)	425 (7.0)	
55–<60	550 (12.2)	931 (15.2)	
60–<65	823 (18.3)	1170 (19.2)	
65–<70	1206 (26.8)	1805 (29.5)	
70–75	1585 (35.2)	1778 (29.1)	
**Sex**			0.042
Male	2688 (59.8)	3513 (57.5)	
Female	1809 (40.2)	2596 (42.5)	
**Charlson co-morbidity index**			<0.001
0	3056 (68.0)	3791 (62.0)	
1–2	1148 (25.5)	1588 (26.0)	
≥3	293 (6.5)	730 (11.9)	
**Smoking status**			<0.001
Active smoker	702 (15.6)	1326 (21.7)	
Former smoker	1679 (37.3)	2146 (35.1)	
Never smoked	1673 (37.2)	2123 (34.8)	
NA	443 (9.9)	514 (8.4)	
**Alcohol consumption (units/week)**			<0.001
None	672 (14.9)	1185 (19.4)	
1–14	2803 (62.3)	3593 (58.8)	
15–21	408 (9.1)	492 (8.1)	
>21	237 (5.3)	366 (6.0)	
NA	377 (8.4)	473 (7.7)	
**BMI (kg/m^2^)**			<0.001
<18.5	200 (4.4)	360 (5.9)	
18.5–25.0	1479 (32.9)	2465 (40.4)	
>25.0–30.0	1766 (39.3)	2076 (34.0)	
>30.0	1052 (23.4)	1208 (19.8)	
**UICC stage**			<0.001
I	1391 (30.9)	835 (13.7)	
II	973 (21.6)	1570 (25.7)	
III	991 (22.0)	1577 (25.8)	
IV	262 (5.8)	994 (16.3)	
Unknown (missing pN)	621 (13.8)	286 (4.7)	
Unknown (other)	259 (5.8)	847 (13.9)	
**Tumour location**			<0.001
Colon	3221 (71.6)	3860 (63.2)	
Caecum	366 (8.1)	698 (11.4)	
Ascending colon	557 (12.4)	774 (12.7)	
Hepatic flexure	173 (3.8)	245 (4.0)	
Transverse colon	230 (5.1)	323 (5.3)	
Splenic flexure	134 (3.0)	163 (2.7)	
Sigmoid colon	1761 (39.2)	1657 (27.1)	
Rectum	1272 (28.3)	2234 (36.7)	
Unspecified	4 (<0.1)	15 (<0.1)	
**Tumour histology**			<0.254
Adenocarcinoma	3190 (70.9)	4814 (78.8)	
Mucoid carcinoma	272 (6.0)	441 (7.2)	
Medullary carcinoma	12 (<0.1)	26 (<0.1)	
Signet ring cell carcinoma	14 (<0.1)	25 (<0.1)	
Unspecified	706 (15.7)	686 (11.2)	
Missing—no tumour left for analysis	301 (6.7)	107 (1.8)	
**Surgical priority**			<0.001
Emergency	36 (0.8)	554 (9.1)	
Elective	4461 (99.2)	5555 (90.9)	

Values are *n* (%). *Chi-squared test or Cochran–Armitage test for trend for ordinal data. NA, not available.

### Intraoperative outcomes

Compared with the NSD-CRC group, rates of intraoperative blood transfusion, tumour perforation, and organ lesion were significantly lower in the SD-CRC group (*Table 2*), whereas the anatomic distribution of organ lesions did not differ between the groups. Intraoperative blood loss was reduced in the SD-CRC group compared with the NDS-CRC group (weighted mean difference (WMD) −51.5 mL, 95% c.i. −66.5 to −36.6, *P* < 0.001).

**Table 2:** Intraoperative and postoperative outcomes for patients with screen-detected colorectal cancer *versus* patients with non-screen-detected colorectal cancer

	Screen-detected colorectal cancer (*n* = 4497)	Non-screen-detected colorectal cancer (*n* = 6109)	RR (95% c.i.)	P*
**Intraoperative outcomes**				
Blood transfusion	37 (0.82)	267 (4.37)	0.29 (0.21,0.41)	<0.001
Tumour perforation	48 (1.06)	308 (4.94)	0.22 (0.16,0.30)	<0.001
Organ lesion	91 (2.02)	197 (3.22)	0.76 (0.59,0.99)	0.042
**Postoperative outcomes**				
Surgical complications	655 (14.78)	1162 (19.02)	0.79 (0.73,0.87)	<0.001
Bowel obstruction	133 (2.96)	278 (4.55)	0.91 (0.74,1.11)	0.354
Superficial wound infection	100 (2.22)	300 (4.91)	0.63 (0.50,0.79)	<0.001
Bleeding	79 (1.76)	153 (2.50)	0.95 (0.72,1.24)	0.681
Fascial dehiscence	56 (1.24)	171 (2.80)	0.58 (0.43,0.78)	0.003
Intra-abdominal abscess	52 (1.16)	186 (3.04)	0.53 (0.39,0.72)	<0.001
Anastomotic leakage†	238 (5.29)	314 (5.14)	1.12 (0.92,1.35)	0.253
Grade A	22 (0.49)	20 (0.33)	1.48 (0.83,2.64)	0.182
Grade B	27 (0.60)	67 (1.10)	0.53 (0.36,0.78)	0.001
Grade C	189 (4.20)	227 (3.72)	1.01 (0.94,1.08)	0.821
Non-surgical complications	284 (6.32)	577 (9.12)	0.68 (0.60,0.78)	<0.001
90-day mortality	47 (1.05)	235 (3.85)	0.29 (0.21,0.39)	<0.001

Values are *n* (%). *Generalized multinomial linear model, adjusted for UICC stage, cancer type, sex, and Charlson co-morbidity index. †Grade A, no management; grade B, therapeutic intervention without re-laparotomy; and grade C, re-laparotomy^27^. RR, relative risk.

After stratified analyses for colon cancer and rectal cancer, rates of blood transfusion (colon cancer: RR 0.32, 95% c.i. 0.21 to 0.49, *P* < 0.001; and rectal cancer: RR 0.49, 95% c.i. 0.18 to 0.63, *P* < 0.001) and tumour perforation (colon cancer: RR 0.12, 95% c.i. 0.07 to 0.19, *P* < 0.001; and rectal cancer: RR 0.55, 95% c.i. 0.37 to 0.83, *P* = 0.004) remained significantly lower in the SD-CRC group than in the NSD-CRC group.

### Postoperative outcomes

Both surgical and non-surgical complications were less frequent in the SD-CRC group than in the NSD-CRC group (*Table 2*). This finding was consistent for surgical complications in patients with colon cancer (RR 0.68, 95% c.i. 0.60 to 0.79, *P* < 0.001) and rectal cancer (RR 0.83, 95% c.i. 0.73 to 0.94, *P* = 0.004). However, a significant reduction in non-surgical complications was only found for patients with colon cancer (RR 0.57, 95% c.i. 0.48 to 0.68, *P* < 0.001). The reduction in the rate of postoperative complications in the SD-CRC group was only observed for patients with UICC stage III, stage IV, or unknown (other) (*Table 3*). A significantly lower rate of non-surgical complications was reported for patients with SD-CRC and operated on in an emergency setting. In the elective setting, the incidences of both surgical and non-surgical complications as well as mortality were reduced in the SD-CRC group (*Table 4*).

**Table 3:** Complications and mortality according to UICC stage for patients with screen-detected colorectal cancer *versus* patients with non-screen-detected colorectal cancer

	Screen-detected colorectal cancer (*n* = 4497)	Non-screen-detected colorectal cancer (*n* = 6109)	RR (95% c.i.)	*P*
**UICC stage I**	1391	835		
Non-surgical complications	85 (6.1)	65 (7.8)	0.81 (0.59,1.11)	0.194
Surgical complications	230 (16.5)	161 (19.3)	0.94 (0.78,1.12)	0.479
90-day mortality	13 (0.9)	10 (1.2)	0.85 (0.37,1.92)	0.709
**UICC stage II**	973	1570		
Non-surgical complications	76 (7.8)	151 (9.6)	0.84 (0.64,1.09)	0.191
Surgical complications	178 (18.3)	288 (18.3)	1.02 (0.87,1.21)	0.784
90-day mortality	9 (0.9)	30 (1.9)	0.56 (0.27,1.19)	0.132
**UICC stage III**	991	1577		
Non-surgical complications	77 (7.8)	144 (9.1)	0.86 (0.66,1.11)	0.241
Surgical complications	146 (14.7)	306 (19.4)	0.77 (0.65,0.92)	0.005
90-day mortality	13 (1.3)	43 (2.7)	0.51 (0.27,0.93)	0.029
**UICC stage IV**	262	994		
Non-surgical complications	19 (7.3)	119 (12.0)	0.58 (0.36,0.92)	0.020
Surgical complications	37 (14.1)	181 (18.2)	0.77 (0.56,1.07)	0.122
90-day mortality	8 (3.1)	117 (11.8)	0.25 (0.12,0.51)	<0.001
**UICC stage unknown (missing pN)**	621	286		
Non-surgical complications	9 (1.4)	8 (2.8)	0.61 (0.23,1.64)	0.330
Surgical complications	69 (11.1)	33 (11.5)	1.25 (0.83,1.88)	0.294
90-day mortality*	–	–	–	–
**UICC stage unknown (other)**	259	847		
Non-surgical complications	18 (6.9)	86 (10.2)	0.64 (0.39,1.04)	0.070
Surgical complications	40 (15.4)	209 (24.7)	0.65 (0.48,0.89)	0.007
90-day mortality*	–	–	–	–

Values are *n* (%) unless otherwise indicated. Generalized multinomial linear model used for all analyses, adjusted for 5-year age group, cancer type, sex, and Charlson co-morbidity index. *Too few data to publish in compliance with European general data protection rules. RR, relative risk.

**Table 4:** Intraoperative and postoperative outcomes stratified into surgical priority (elective or emergency) for patients with screen-detected colorectal cancer *versus* patients with non-screen-detected colorectal cancer

	Screen-detected colorectal cancer (*n* = 4497)	Non-screen-detected colorectal cancer (*n* = 6109)	RR (95% c.i.)	*P*
**Elective surgery**	4461	5555		
Intraoperative outcomes				
Organ lesion	91 (2.0)	172 (3.1)	0.74 (0.57,0.96)	0.024
Blood transfusion	37 (0.8)	222 (4.0)	0.30 (0.21,0.43)	<0.001
Tumour perforation	39 (0.9)	171 (3.1)	0.30 (0.24,0.49)	<0.001
Postoperative outcomes				
Surgical complications	647 (14.5)	1042 (18.8)	0.81 (0.74,0.88)	<0.001
Non-surgical complications	280 (6.3)	438 (7.9)	0.82 (0.71,0.95)	0.007
90-day mortality	39 (0.9)	123 (2.2)	0.39 (0.27,0.56)	<0.001
**Emergency surgery**	36	554		
Intraoperative outcomes				
Organ lesion	0 (0)	25 (4.5)	–	–
Blood transfusion	0 (0)	45 (8.1)	–	–
Tumour perforation	9 (25.0)	137 (24.7)	1.14 (0.67,2.04)	0.586
Postoperative outcomes				
Surgical complications	8 (22.2)	120 (21.7)	1.07 (0.67,2.04)	0.822
Non-surgical complications	3 (8.3)	149 (26.9)	0.34 (0.07,0.99)	0.049
90-day mortality	8 (22.2)	112 (20.2)	1.35 (0.77–2.56)	0.350

Values are *n* (%) unless otherwise indicated. Generalized multinomial linear model used for all analyses, adjusted for 5-year age group, cancer type, sex, and Charlson co-morbidity index. RR, relative risk.

The specific types of surgical complications with a lower incidence in the SD-CRC group were fascial dehiscence, superficial wound infection, and intra-abdominal abscess. There were no significant differences between the two groups in the rates of postoperative bleeding, bowel obstruction, or anastomotic leakage. However, grade B anastomotic leakage was more frequent in the NSD-CRC group (RR 0.53, 95% c.i. 0.36 to 0.78, *P* = 0.001). After surgery, patients with SD-CRC were characterized by significantly lower Clavien–Dindo grades (*Table 5*).

**Table 5:** Complications expressed as highest Clavien–Dindo grade for patients with screen-detected colorectal cancer *versus* patients with non-screen-detected colorectal cancer

	Screen-detected colorectal cancer (*n* = 4497)	Non-screen-detected colorectal cancer (*n* = 6109)	P*
**Clavien–Dindo grade**			<0.001
0	3698 (82.23)	4595 (75.22)	
I	110 (2.45)	194 (3.18)	
II	160 (3.56)	293 (4.80)	
IIIa	78 (1.73)	209 (3.42)	
IIIb	310 (6.89)	539 (8.82)	
IVa	67 (1.49)	115 (1.88)	
IVb	25 (0.56)	47 (0.77)	

Values are *n* (%). *Cochran–Armitage test for trend. Excluded from analysis due to missing data or death: screen-detected colorectal cancer, *n* = 49; and non-screen-detected colorectal cancer, *n* = 117.

The duration of hospitalization was significantly lower for patients with SD-CRC (mean of 8.33 days) than patients with NSD-CRC (mean of 11.33 days) (WMD −5.40 days, 95% c.i. −7.83 to −2.97). Besides SD-CRC screening status, a shorter duration of hospitalization was independently predicted by colon cancer, female sex, and CCI 0 *versus* greater than or equal to 3 (*Table 6*).

**Table 6:** Prediction of duration of hospitalization

	Duration of hospitalization (days), mean (95% c.i.)	P*
**Screening status**		
Screen-detected colorectal cancer	−2.32 (−2.81,−1.82)	<0.001
Non-screen detected colorectal cancer	0	–
**Type of cancer**		
Colonic	−3.99 (−4.50,−3.48)	<0.001
Rectal	0	–
**Age (years)**		
50–<55	−0.19 (−1.11,0.73)	0.688
55–<60	−0.08 (−0.87,0.72)	0.851
60–<65	−0.07 (−0.76,0.62)	0.848
65–<70	−0.04 (−0.65,0.58)	0.905
70–75	0	–
**Sex**		
Female	−0.744 (−1.22,−0.26)	0.002
Male	0	–
**Charlson co-morbidity index**		
0	−0.93 (−1.69,−0.17)	0.017
1–2	−0.35 (−1.19,0.48)	0.408
≥3	0	–

*Multivariable generalized linear regression model.

The 90-day overall mortality was significantly reduced by 71.2% in the SD-CRC group. This reduction remained statistically significant when additionally adjusting for surgical priority (emergency/elective). Significantly lower 90-day mortality in the SD-CRC group was observed for both patients with colon cancer (RR 0.23, 95% c.i. 0.16 to 0.30, *P* < 0.001) and patients with rectal cancer (RR 0.41, 95% c.i. 0.23 to 0.73, *P* = 0.002). After stratification for UICC stage, the reduced 90-day mortality was only significant for UICC stages III and IV (*Table 3*).

## Discussion

This study shows improved surgical course and outcomes, including lower 90-day mortality, in patients with SD-CRC compared with patients with NSD-CRC. Even after adjustment for the most important confounders, both intraoperative and postoperative outcomes are significantly improved in the SD-CRC group compared with the NSD-CRC group. This is the case for both surgical and non-surgical complications.

Treatment of SD-CRC is associated with an approximate 70% reduction in 90-day mortality. Some of this difference can be explained by a lower UICC stage in the SD-CRC group at the time of diagnosis. However, sub-analyses reveal that this between-group difference is only significant in patients with UICC stage III or stage IV disease. There are three possible explanations for this. First, patients with SD-CRC might have less advanced disease compared with patients with NSD-CRC within the same UICC stage. This difference might be larger in UICC stages III and IV than in stages I and II. Second, patients with SD-CRC could have more favourable demographic and disease-related characteristics other than were adjusted for in the present analyses. Third, UICC stage I and II patients had very low mortality, increasing the risk of a statistical type 2 error concerning this outcome.

The 90-day mortality is reduced in the SD-CRC group—both for colon cancer and rectal cancer. A large Dutch study found reduced 30-day mortality only in patients with screen-detected colon cancer UICC stages I to III and in patients with screen-detected rectal cancer UICC stage IV^16^. de Neree Tot Babberich *et al*.^16^ reported on 30-day mortality, whereas the present study reports on 90-day mortality, which might contribute to the disagreement in the results. Because the sample size and number of events in the present study did not allow for stratification for both UICC and location of cancer in one analysis, and the two studies differed with regard to length of follow-up, it is difficult to conclude which subpopulation with regard to cancer location benefits the most from CRC screening.

There are four previous studies that compared SD-CRC and NSD-CRC^15–18^. In agreement with the present study, three of these reported a lower rate of surgical complications in the SD-CRC group^15–17^. The most well-powered study additionally revealed significantly lower rates of non-surgical complications in the SD-CRC group across UICC stages I to III for patients with colon cancer^16^. Correspondingly, the present study only found lower rates of both surgical and non-surgical complications for patients with SD-CRC colon cancer in UICC stages III and IV, as the rate of non-surgical complications in patients with screen-detected rectal cancer was not significantly reduced. In addition, the prevalence of rectal cancer is lower in screened populations^15,17,18^, partly because patients with rectal cancer are more symptomatic than patients with colon cancer^32^. Taken together, these findings suggest a greater benefit from CRC screening for patients with colon cancer.

Two studies^15,18^ were considerably smaller than the present study and might therefore be less well powered. The two others assessed healthy user bias by adjustment for BMI in combination with ASA physical status classification^17^ or BMI, ASA physical status classification, and CCI^16^. Besides age, sex, and location of cancer, all analyses in the present study were adjusted for CCI, as CCI is considered a more appropriate measurement for healthy user bias^33,34^. There is at this point no consensus on how to best adjust for the complex phenomenon of healthy user bias.

There is evidence that the rate of open surgery for patients with SD-CRC is lower^16–18^. A study conducted on the same population reported a significantly higher chance of minimally invasive surgery for patients with SD-CRC (RR 1.16) and this difference was consistent across all UICC stages^35^. This surgical practice would be expected to contribute to a reduction in postoperative complications and mortality^19–21^. Most SD-CRC tumours never result in symptoms, reducing the need for emergency surgeries. Because most emergency surgeries are open and more often have a complicated course, this might be part of the explanation for why patients with SD-CRC have better surgical outcomes, in particular those with cancer in UICC stages III to IV.

There is no overall difference in the rates of anastomotic leakages between the two populations, except for a lower rate of grade B anastomotic leakages in patients with SD-CRC. The authors have no good explanation for this and it might be the result of type one statistical error.

Not surprisingly, treatment of SD-CRC is associated with shorter hospitalization^36,37^ and less intraoperative bleeding. Although the clinical relevance of reporting differences in intraoperative blood loss is questionable due to imprecise and inconsistent assessment^38,39^, the present study also showed less blood transfusion in the SD-CRC group. The NSD-CRC group is characterized by higher rates of advanced malignant disease, open surgery, organ lesion, and tumour perforation. All these factors can potentially lead to more bleeding and other surgical complications.

The present study was strengthened by its nationwide design and the efforts to adjust for length and healthy user bias^23^. However, there were limitations. Missing data were encountered, but, because of a large population and equal distribution between the groups, it is assumed that this factor did not substantially affect the relative outcome risks. As screening impacted the UICC stage, it was not possible to adjust for this factor in the analyses. However, UICC stage-stratified sub-analyses on the outcomes were conducted to overcome that. While adjustment for some of the confounders was performed, it was not possible to adjust for all and therefore the results might be affected by residual healthy user bias. Moreover, the present study did not consider if patients had had prior surgery, which may lead to more complications^40^.

The data in the present study are from the first round of CRC screening in Denmark; the distribution of patient and tumour characteristics will likely change during the second and third rounds of screening. Less advanced UICC stages are thus expected for patients with SD-CRC in future screening rounds, which in turn might lead to even better outcomes for the patients with SD-CRC compared with patients with NSD-CRC^41^.

In conclusion, the implementation of a nationwide, publicly funded, FIT-based CRC screening programme leads to more favourable postoperative outcomes, with shortened hospitalization and reduced rates of complications and short-term mortality—even after adjustment for potential healthy user bias.

## Data Availability

Due to Danish data-protection law, data cannot be made publicly available.
